# Investigating changes in mental illness stigma and discrimination after the Time to Change programme in England

**DOI:** 10.1192/bjo.2024.801

**Published:** 2024-11-06

**Authors:** Amy Ronaldson, Claire Henderson

**Affiliations:** Centre for Implementation Science, Health Service and Population Research Department, Institute of Psychiatry, Psychology, and Neuroscience, King's College London, UK

**Keywords:** Mental health, stigma and discrimination, Time to Change, social distance, population mental health

## Abstract

**Background:**

Between 2008 and 2019, we reported positive change relating to mental health stigma and discrimination among the adult population of England, supporting the effectiveness of the Time to Change campaign.

**Aims:**

Using data from the Attitudes to Mental Illness survey (2008/2009 to 2023), we investigated the extent to which positive changes in stigma were sustained by 2023, 2 years after the programme's end in 2021.

**Method:**

We used regression analyses to evaluate trends in outcomes. Measures were of stigma-related knowledge (Mental Health Knowledge Schedule (MAKS)), attitudes (Community Attitudes toward the Mentally Ill scale (CAMI)) and desire for social distance (Reported and Intended Behaviour Scale (RIBS)). We also examined willingness to interact with people based on vignettes of depression and schizophrenia, and attitudes toward workplace discrimination, using data from the British Social Attitudes Survey for comparison.

**Results:**

CAMI scores improved between 2008 and 2023 (s.d. 0.24, 95% CI 0.16–0.31), but decreased since 2019 (*P* = 0.015). After improvements between 2009 and 2019, 2023 MAKS and RIBS scores no longer differed from 2009 scores, indicating decreases in stigma-related knowledge (MAKS scores declined 7.8%; *P* < 0.001) and willingness to interact (RIBS scores declined by 10.2%; *P* < 0.001) since 2019. Conversely, comparison with British Social Attitudes Survey data indicated that willingness to interact with people with depression and schizophrenia increased gradually between 2007, 2015 and 2023, and attitudes to workplace discrimination also improved.

**Conclusions:**

The lasting positive changes reflect support for non-discrimination and willingness to interact with someone after a sense of familiarity is evoked. Besides the end of Time to Change, interpretations for declines in other outcomes include the COVID-19 pandemic and economic stress.

Stigma and discrimination against people with mental illness have substantial public health impact, contributing to inequalities^1^ such as poor access to mental and physical healthcare,^2^ reduced life expectancy,^3^ exclusion from higher education and employment,^4^ increased contact with criminal justice systems, victimisation,^5^ poverty and homelessness. There is growing investment in and evidence for the effectiveness of anti-stigma interventions, including programmes targeted at the general population and/or specific groups.^6^

## Time to change

In England, a programme against stigma and discrimination, Time to Change,^7^ was delivered by the charities Mind and Rethink Mental Illness between 2007 and 2021. Its first two phases ran from 2007 to 2011 and 2011 to 2015, and included a social marketing campaign launched in January 2009 that was aimed at adults aged 25–44 years in middle-income groups, and work with target groups. To evaluate Time to Change's effect on public stigma, in 2009, measures of stigma-related knowledge and desire for social distance were added to the pre-existing national Attitudes to Mental Illness survey (AMI).^8^

Between 2009 and 2019, there were significant improvements in stigma-related knowledge, attitudes and desire for social distance.^9^ Surveys of those who used mental health services showed evidence for a reduction in direct experiences of discrimination across multiple areas of life, particularly informal relationships, between 2008 and 2014.^10^

Time to Change phase 3 ran from 2016 to 2021. The social marketing campaign from 2017 was aimed again at those aged 25–44 years, but in an income group overlapping but lower than before, and focused on men's mental health. The campaign promoted empathy as a key mediator of the effect of contact on prejudice,^11^ while encouraging people to maintain contact^12^ (as opposed to avoidance). In the process, the campaign delivered parasocial (virtual) contact^12^ and promoted imagined contact.^13^ Time to Change ended on 31 March 2021.

We previously reported positive change in stigma-related knowledge, attitudes to mental illness and desire for social distance from people with mental illness among the adult general population of England between 2008 and 2019, supporting the effectiveness of the social marketing campaign.^9^

Other research conducted during the COVID-19 pandemic^14^ found an increase in desire for social distance from people with mental illness between March 2020 and March 2022, together with a decline in other measures designed to capture aspects of mental health literacy.^15^

## Study aims

We therefore investigated the extent to which the changes reported from 2008 to 2019 were sustained by 2023, 2 years after the end of Time to Change in England. We also wished to address a limitation of the measures, which enquire about mental illness or mental health problems in general, since research in other countries has shown differential changes over time in desire for social distance for depression versus schizophrenia.^16^ Most recently, research in the USA suggests decreased desire for social distance from people with depression between 2006 and 2018, but a possible slight increase regarding people with schizophrenia, along with increased perception of dangerousness.^17^ In the UK, different patterns of change in newspaper coverage between schizophrenia and other disorders have been observed suggesting a relative lack of improvement in coverage about people with schizophrenia.^18^ Therefore, we also compared attitudes and desire for social distance toward people with depression and schizophrenia in 2023 with those available from other time points during Time to Change, by repeating measures used in the 2007 and 2015 British Social Attitudes Survey (BSAS).^19^

## Method

### Data source

**Fig. 1:**
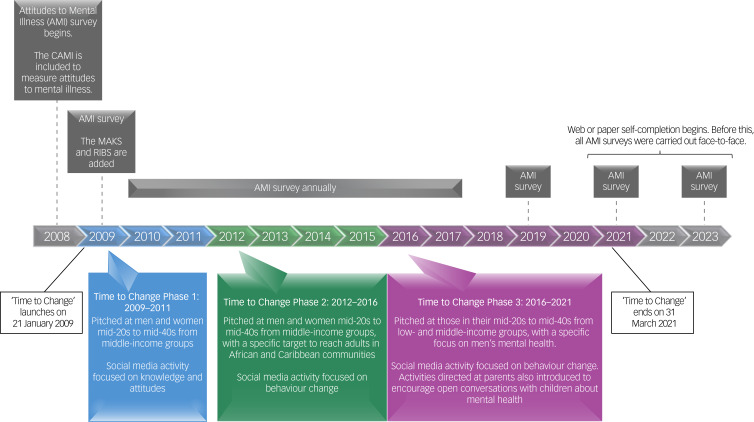
A timeline of Time to Change and the Attitudes to Mental Illness surveys. AMI, Attitudes to Mental Illness survey; CAMI, Community Attitudes toward the Mentally Ill scale; MAKS, Mental Health Knowledge Schedule; RIBS, Reported and Intended Behaviour Scale.

The AMI was carried out annually in England from 2008 to 2017, every 2 years since 2017 by Kantar TNS, and previously intermittently from 1994. Approximately 1700 respondents take part. A quota sampling frame is used to ensure a nationally representative sample of adults (16 years or older) living in England who could be exposed to the Time to Change campaign, and respondents are not resampled in later surveys. Detailed information about sampling methods^20^ can be obtained via the authors. Until 2019, respondents were interviewed face to face at home, by trained personnel. Since then, data have been collected using address-based online surveying (https://www.kantar.com/-/media/Project/Kantar/Global/Expertise/Policy-and-Society/Address-Based-Online-Surveying.PDF), which offers web or paper self-completion. Measures of stigma-related knowledge and desire for social distance were added in 2009, just before the Time to Change social marketing campaign launched; therefore, the baseline for attitudes is 2008, and for the other outcomes is 2009. A detailed timeline of the AMI and the Time to Change campaign is provided in Fig. 1.

### Measures

#### Stigma-related knowledge

Stigma-related knowledge was measured with the first six items of the Mental Health Knowledge Schedule (MAKS),^21^ covering help-seeking, recognition, support, employment, treatment and recovery (e.g. ‘Most people with mental health problems want to have paid employment’). The standardised total score was used. These items were rated on a scale ranging from 1 (strong disagreement) to 5 (strong agreement), with higher scores indicating greater knowledge. The Cronbach's alpha for the total scale in the current study was 0.59. This relatively low internal consistency has been reported previously,^9^ and likely reflects the different knowledge domains measured by each item.^21^

#### Attitudes to mental illness

Attitudes to mental illness were measured with 26 of the 40 items of the Community Attitudes toward the Mentally Ill scale (CAMI),^22^ plus an item on employment-related attitudes added when the UK Department of Health first commissioned the survey in 1994.^8^ The CAMI has two factors: ‘Prejudice and Exclusion’ (e.g. ‘One of the main causes of mental illness is a lack of self- discipline and willpower’) and ‘Tolerance and Support for Community Care’ (e.g. ‘We need to adopt a far more tolerant attitude toward people with mental illness in our society’).^23^ All items were rated on a scale ranging from 1 (strong disagreement) to 5 (strong agreement). The standardised scores of the total CAMI, ‘Prejudice and Exclusion’ and ‘Tolerance and Support for Community Care’ subscales were used. Higher scores indicate less stigmatising attitudes to mental illness. The internal consistency of the CAMI in this data-set was 0.87 (Cronbach's alpha) for the total scale, and 0.85 and 0.76 for ‘Prejudice and Exclusion’ and ‘Tolerance and Support for Community Care’, respectively.

#### Desire for social distance

Desire for social distance was measured with the four items of the Reported and Intended Behaviour Scale (RIBS),^24^ which constitute the Intended Behaviour subscale and assess desire for social distance in terms of living with, working with, living nearby and continuing a relationship with someone with a mental illness. These items were rated on a scale ranging from 1 (strong disagreement) to 5 (strong agreement), with higher scores indicating less desire for social distance. The total score was standardised. The internal consistency of the four items was 0.84 (Cronbach's alpha).

#### Measures from the BSAS 2007 and 2015

For the first time in the AMI, we included items used in the 2007 and 2015 BSAS conducted by the National Centre for Social Research (https://natcen.ac.uk/british-social-attitudes). This annual survey is designed to produce a representative sample of adults aged 18 years or over, who are living in private households. These data were accessed directly from the UK Data Service (https://beta.ukdataservice.ac.uk/datacatalogue/series/series?id=200006). We included BSAS participants living in England who responded to vignettes of a common mental health problem (depression) and a less common problem (schizophrenia). This allowed us to compare desire for social distance between 2007 (BSAS, considered to be baseline), 2015 (BSAS) and 2023 (AMI). Two different people were described, and respondents were asked how willing they would be to interact with them in a range of situations (e.g. spend time socialising, move next door to). In 2007, the survey design randomly allocated respondents to a depression or schizophrenia vignette. In 2015 and 2023, the entire sample responded. The vignettes were not labelled ‘depression’ or ‘schizophrenia’. People were asked social distance questions for each vignette about willingness to have the person in the vignette as a neighbour, to socialise with them, to have them care for their children, to befriend them, to work with them and to have them marry someone they know. In 2023 and 2015, participants responded to these items on a five-point Likert type scale, with 1 being very willing, 2 being fairly willing, 3 being neither willing nor unwilling, 4 being fairly unwilling and 5 being very unwilling. In 2007, participants responded on a four-point Likert type scale, with 1 being definitely willing, 2 being probably willing, 3 being probably unwilling and 4 being definitely unwilling. Using BSAS 2015 data, we also repeated questions assessing expectations of and attitudes toward workplace discrimination against people with depression and schizophrenia compared with a physical health comparator (diabetes). Specifically, respondents were asked about the likelihood of people with these conditions to be promoted, who had had repeated periods of time off work but whose illness was now under control through medication, and were asked whether medical history should make a difference. Attitudes towards workplace discrimination were not measured in 2007.

#### Familiarity with someone with a mental health problem

Familiarity with someone with a mental health problem was measured with the item: ‘Who is the person closest to you who has or has had some kind of mental illness?’. Responses included self, immediate family, partner, other family, friend, acquaintance, work colleague, other or no-one. The response to this item was categorised into three groups: self, other or none.

#### Sociodemographic variables

Several sociodemographic variables were included in the analysis: age, gender, ethnicity (Asian, Black, Other, White), socioeconomic position and government office region. Socioeconomic position was based on the chief income earner of each household, using the Market Research Society's classification system (AB, C1, C2, DE). AB represents professional /managerial occupations, C1 represents other non-manual occupations, C2 represents skilled manual occupations and DE represents semi-/unskilled manual occupations and people dependent on state benefits. Government office region is the lowest level information on participants’ location as described by the UK Government's Office for National Statistics (ONS) (see Table 1).

**Table 1:** Participant demographics by survey year, unweighted frequency and weighted per cent

	2008 (*n* = 1703)	2009 (*n* = 1751)	2010 (*n* = 1745)	2011 (*n* = 1741)	2012 (*n* = 1717)	2013 (*n* = 1727)	2014 (*n* = 1714)	2015 (*n* = 1736)	2016 (*n* = 1765)	2017 (*n* = 1720)	2019 (*n* = 1785)	2021 (*n* = 1471)	2023 (*n* = 1638)
Gender, *n* (%)													
Female	925 (51.7	939 (51.5)	939 (51.7)	912 (51.5)	924 (51.3)	926 (51.0)	893 (50.9)	919 (51.6)	918 (51.4)	938 (50.8)	933 (51.1)	877 (50.9)	1002 (51.3)
Male	778 (48.3)	812 (48.5)	806 (48.3)	829 (48.5)	793 (48.7)	801 (49.0)	821 (49.1)	817 (48.4)	847 (48.6)	782 (49.2)	852 (48.9)	570 (49.1)	606 (48.7)
Age, years, mean (s.d.)	46.7 (18.9)	46.0 (18.8)	46.5 (18.4)	46.4 (19.2)	46.4 (19.1)	45.9 (18.3)	46.0 (18.8)	46.4 (19.2)	46.3 (19.7)	43.7 (20.0)	46.0 (20.4)	Not available	Not available
Age group, years, *n* (%)													
16–24	188 (13.2)	247 (14.3)	240 (14.6)	235 (14.4)	258 (14.6)	289 (14.6)	221 (14.4)	242 (14.1)	211 (13.6)	220 (17.8)	275 (14.5)	215 (12.9)	142 (12.8
25–44	562 (36.0)	633 (35.9)	540 (35.1)	545 (35.4)	580 (34.8)	568 (36.1)	514 (36.2)	528 (35.3)	597 (35.5)	491 (37.4)	521 (36.2)	522 (33.2)	496 (32.6)
45–64	525 (32.1)	512 (31.3)	549 (31.5)	499 (30.6)	506 (31.3)	486 (31.1)	506 (30.6)	488 (31.5)	488 (31.7)	507 (27.7)	484 (30.9)	402 (32.3)	491 (31.8)
≥65	428 (18.7)	359 (18.5)	416 (19.4)	462 (19.5)	373 (19.3)	384 (18.3)	473 (18.7)	478 (19.0)	469 (19.3)	502 (17.1)	505 (18.5)	302 (21.6)	508 (22.8)
Ethnicity, *n* (%)													
Asian	90 (5.5)	112 (6.2)	136 (8.5)	134 (8.1)	160 (9.7)	127 (7.9)	105 (6.6)	120 (6.7)	121 (7.0)	76 (5.5)	97 (6.5)	117 (8.4)	93 (8.5)
Black	73 (4.4)	63 (3.4)	88 (4.9)	64 (3.8)	67 (3.8)	66 (3.7)	69 (4.0)	99 (5.3)	83 (4.7)	61 (4.5)	82 (4.7)	21 (1.4)	29 (2.0)
Other	28 (1.9)	26 (1.4)	18 (1.1)	20 (1.1)	31 (1.8)	44 (2.6)	26 (1.6)	39 (2.3)	42 (2.6)	41 (3.1)	57 (3.7)	29 (1.9)	54 (4.5)
White	1503 (88.1)	1542 (89.0)	1496 (85.5)	1504 (87.0)	1449 (84.7)	1474 (85.9)	1507 (87.8)	1472 (85.7)	1507 (85.7)	1529 (87.0)	1535 (85.1)	1281 (88.3)	1423 (85.0)
Socioeconomic position, Market Research Society's classification system, *n* (%)													
AB	315 (21.0)	279 (19.4)	300 (20.2)	322 (20.5)	292 (19.3)	302 (20.5)	353 (21.4)	335 (22.2)	271 (18.9)	350 (22.4)	291 (20.5)	541 (39.8)	593 (39.1)
C1	433 (29.6)	454 (32.2)	464 (31.7)	450 (29.8)	456 (31.0)	445 (30.4)	457 (29.2)	432 (28.4)	430 (30.6)	501 (35.3)	443 (30.4)	317 (21.8)	308 (19.7)
C2	363 (21.1)	389 (20.8)	342 (19.2)	340 (21.1)	368 (21.6)	362 (20.8)	333 (20.5)	354 (20.4)	371 (20.7)	296 (15.4)	383 (20.9)	181 (12.1)	195 (13.0)
DE	592 (28.2)	629 (27.6)	639 (28.8)	629 (28.6)	601 (28.1)	618 (29.1)	571 (29.0)	615 (29.1)	693 (29.8)	573 (26.9)	668 (28.3)	423 (26.4)	520 (28.2)
Familiarity with mental health problems, *n* (%)													
Self	102 (6.0)	92 (5.0)	75 (4.2)	90 (5.6)	111 (6.4)	120 (6.6)	126 (7.4)	124 (6.9)	124 (7.4)	143 (9.2)	152 (8.9)	253 (17.5)	229 (15.4)
Other	665 (42.5)	902 (54.0)	892 (53.0)	896 (53.5)	926 (55.9)	963 (57.9)	953 (57.5)	963 (58.1)	1013 (61.1)	980 (58.8)	929 (55.0)	953 (70.4)	1076 (69.9)
None	846 (51.5)	718 (41.0)	738 (42.8)	706 (41.0)	645 (37.7)	610 (35.5)	606 (35.1)	632 (35.0)	586 (31.5)	566 (32.0)	659 (36.1)	150 (12.2)	232 (14.7)
Region, *n* (%)													
North-East	86 (5.3)	86 (5.0)	88 (4.8)	83 (4.8)	95 (5.5)	82 (4.9)	76 (4.4)	76 (5.5)	98 (5.3)	73 (4.6)	86 (4.7)	82 (4.8)	104 (4.9)
North-West	218 (12.9)	245 (14.2)	244 (13.6)	233 (13.6)	235 (12.8)	233 (13.6)	240 (13.8)	280 (20.5)	242 (13.8)	238 (13.6)	250 (13.2)	172 (12.9)	248 (12.9)
York and Humber	172 (9.8)	174 (9.6)	178 (9.9)	176 (10.5)	185 (10.3)	174 (9.9)	169 (10.6)	131 (9.6)	186 (10.3)	142 (7.9)	188 (10.7)	156 (9.7)	168 (9.7)
East Midlands	157 (9.1)	151 (8.7)	147 (9.9)	159 (8.5)	129 (8.2)	152 (8.6)	140 (8.0)	139 (7.2)	143 (8.1)	152 (7.8)	154 (9.0)	120 (8.6)	148 (8.6)
West Midlands	173 (9.7)	192 (10.9)	190 (10.5)	195 (10.4)	186 (10.9)	188 (10.8)	189 (10.6)	167 (8.6)	189 (10.6)	188 (11.0)	173 (9.7)	145 (10.5)	145 (10.4)
East	186 (11.3)	187 (10.4)	193 (11.3)	192 (10.6)	178 (10.3)	188 (11.0)	202 (11.3)	202 (10.5)	191 (11.3)	199 (10.8)	196 (11.7)	156 (11.1)	204 (11.1)
South-East	287 (16.9)	278 (16.7)	282 (17.0)	285 (16.9)	291 (17.6)	285 (16.0)	287 (17.1)	305 (16.0)	290 (16.9)	295 (17.1)	283 (15.6)	243 (16.3)	263 (16.3)
South-West	161 (9.1)	176 (10.1)	173 (9.6)	176 (10.5)	170 (10.1)	179 (10.4)	164 (9.8)	146 (7.3)	176 (10.0)	181 (10.5)	171 (9.0)	198 (10.1)	215 (10.1)
London	263 (16.0)	262 (14.5)	250 (14.8)	248 (14.4)	248 (14.4)	246 (14.9)	247 (14.6)	290 (14.6)	250 (13.8)	252 (16.8)	284 (16.4)	199 (16.0)	143 (16.0)

### Statistical analysis

Descriptive statistics for participant demographics and crude outcome scores were calculated and reported by survey year. All analyses were weighted by age, gender and ethnicity, to reflect population characteristics in England. Survey weights were taken from the ONS. For modelling, the quota sample was treated as a probability sample.

We used multiple regression models to evaluate patterns of change in (a) stigma-related knowledge (MAKS scores), (b) attitudes to mental illness (CAMI scores) and (c) desire for social distance (RIBS Intended Behaviour score). All models used the standardised scores of the measures as dependent variables, meaning the outputs were interpreted in standard deviation units. All the models included a fixed effect for year, using a categorical dummy variable. We used the distributional approach to obtain estimates for the proportion of the population whose outcomes changed between two comparative years (i.e. 2023 and baseline, 2023 and 2019).^25^ This method uses the parameters of the normal distribution and converts results from linear regression models into corresponding proportions, using the area under the standard normal curve. We included covariates to control for differences in sociodemographic factors: age group, gender, ethnicity, socioeconomic position, familiarity with someone with a mental health problem (self, other, none) and government office region. The same analysis has been applied to previous AMI data,^9,26–28^ except that the government office region variable was not included during phase 1 of Time to Change.^28^ Interactions between year and sociodemographic factors (age group, gender, ethnicity, socioeconomic position, government office region) were tested to see whether patterns of change in outcomes over time differed by groups. The interaction terms were added separately to the initial models and evaluated for statistical significance with a Wald test.

To compare desire for social distance (vignettes) and attitudes toward workplace discrimination, we merged and harmonised relevant data from the BSAS 2007 and 2015 with data from the AMI survey 2023. Logistic regression models were used to assess change between 2007, 2015 and 2023 in willingness to interact with a person with depression (‘Stephen’) or schizophrenia (‘Andy’). Data from 2007 acted as the reference. Because of different response categories in 2007 versus 2015/2023, a binary outcome variable was created; ‘1’ indicated that someone was fairly (probably) or very (definitely) willing, and ‘0’ indicated someone was fairly (probably) or very (definitely) unwilling. Responses that indicated someone was neither willing nor unwilling were excluded from this analysis to facilitate comparison across years. Sociodemographic factors common to both datasets were included as covariates in the logistic regression models: age group, gender, ethnicity, socioeconomic position and government office region.

Similarly, logistic regression models were used to assess changes in attitudes toward workplace discrimination between 2015 and 2023. A binary outcome was created relating to likelihood of promotion for each condition, where ‘1’ indicated that a person would be just as likely and ‘0’ indicated that they would be slightly or much less likely. A binary outcome was also created relating to whether someone's condition should make a difference to likelihood of promotion, with ‘1’ indicating that they definitely/probably should and ‘0’ indicating that they probably/definitely should not.

All analyses were performed with Stata version 17.0 for Windows (Stata Corp, College Station, Texas, USA).

### Ethics

The King's College London Psychiatry, Nursing and Midwifery Research Ethics Subcommittee exempted analysis of these survey data as secondary analysis of anonymised data.

## Results

### Sample characteristics

Participant demographics are reported by survey year in Table 1. Over time, the distribution of male and female participants and their regional distribution remains stable. Since the introduction of remote data collection (2021), there have been changes in some sociodemographic factors: more participants were 45 years or older, had professional/managerial occupations and had experience with mental health problems (self and other). In 2021 and 2023, fewer Black respondents took part.

### Attitudes to mental illness

**Fig. 2:**
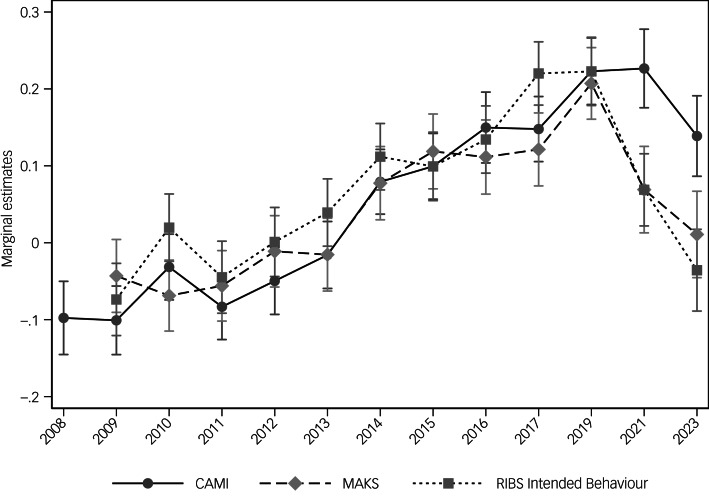
Marginal estimates of stigma-related attitudes (CAMI), knowledge (MAKS) and desire for social distance (RIBS intended behaviour), by year (95% confidence intervals). CAMI, Community Attitudes toward the Mentally Ill scale; MAKS, Mental Health Knowledge Schedule; RIBS, Reported and Intended Behaviour Scale.

Figure 2 depicts the change over time in total CAMI scores by plotting the marginal estimates of the standardised scores by year. There have been improvements in total CAMI scores since 2008 (see Table 2). In 2023, participants scored 0.24 (95% CI 0.16–0.31) s.d. units higher on the CAMI scale than in 2008, translating to a 9.4% improvement in attitudes (*P* < 0.001). However, between 2019 and 2023, total CAMI scores declined by 3.3% (*P* = 0.015). An interaction between year and both age and government region (adjusted Wald tests *P* < 0.001) suggests that changes in CAMI scores over time differ according to these sociodemographic factors (Supplementary Figs 1 and 2 available at https://doi.org/10.1192/bjo.2024.801). Declines in scores since 2019 are most pronounced in those aged 45–64 years and in those residing in South-East England.

**Table 2:** Multiple linear regression analyses of predictors of mental health-related attitudes, knowledge and behaviour among the general public

Predictors	Attitudes: standardised CAMI scores (*n* = 21 334)		Knowledge: standardised MAKS scores (*n* = 19 726)		Intended behaviour: standardised RIBS Intended Behaviour subscale scores (*n* = 19 726)	
	Standardised effect size (95% CI)	*P*-value	Standardised effect size (95% CI)	*P*-value	Standardised effect size (95% CI)	*P*-value
Year						
2023	0.24 (0.16–0.31)*	<0.001	0.05 (−0.02 to 0.13)	0.153	0.04 (−0.03 to 0.11)	0.294
2021	0.32 (0.25–0.39)*	<0.001	0.11 (0.04−0.19)*	0.003	0.14 (0.07−0.21)*	<0.001
2019	0.32 (0.26–0.38)*	<0.001	0.25 (0.18−0.32)*	<0.001	0.30 (0.23−0.36)*	<0.001
2017	0.24 (0.18–0.31)*	<0.001	0.16 (0.09−0.23)*	<0.001	0.29 (0.23−0.36)*	<0.001
2016	0.25 (0.18–0.32)*	<0.001	0.15 (0.09−0.22)*	<0.001	0.21 (0.14−0.27)*	<0.001
2015	0.20 (0.13–0.26)*	<0.001	0.16 (0.09−0.23)*	<0.001	0.17 (0.11−0.24)*	<0.001
2014	0.18 (0.11–0.24)*	<0.001	0.12 (0.05−0.19)*	<0.001	0.18 (0.12−0.25)*	<0.001
2013	0.08 (0.02–0.15)*	0.013	0.03 (−0.04 to 0.09)	0.418	0.11 (0.05−0.18)*	0.001
2012	0.05 (−0.02 to 0.11)	0.144	0.03 (−0.03 to 0.10)	0.344	0.07 (0.01−0.14)*	0.024
2011	0.01 (−0.05 to 0.08)	0.656	−0.01 (−0.08 to 0.05)	0.700	0.03 (−0.04 to 0.09)	0.392
2010	0.07 (0.00–0.13)*	0.042	−0.02 (−0.09 to 0.04)	0.447	0.09 (0.03−0.16)*	0.004
2009 (reference)	−0.00 (−0.07 to 0.06)	0.925	−	−	−	−
2008 (reference CAMI)	−	−	−	−	−	−
Gender						
Female	0.17 (0.15–0.20)*	<0.001	0.16 (0.13−0.18)*	<0.001	−0.00 (−0.03 to 0.02)	0.870
Male (reference)	−	−	−	−	−	−
Age, years						
16–24	0.03 (−0.01 to 0.07)	0.206	0.03 (−0.01 to 0.08)	0.179	0.53 (0.49−0.58)*	<0.001
25–44	0.12 (0.08–0.15)*	<0.001	0.15 (0.11−0.18)*	<0.001	0.45 (0.42−0.49)*	<0.001
45–64	0.21 (0.17–0.24)*	<0.001	0.22 (0.18−0.26)*	<0.001	0.40 (0.36−0.44)*	<0.001
≥65 (reference)	−	−	−	−	−	−
Ethnicity						
Asian	−0.42 (−0.47 to −0.37)*	<0.001	−0.08 (−0.13 to −0.02)*	0.005	−0.40 (−0.47 to −0.34)*	<0.001
Black	−0.35 (−0.41 to −0.28)*	<0.001	−0.02 (−0.10 to 0.05)	0.502	−0.25 (−0.33 to −0.17)*	<0.001
Other	−0.27 (−0.36 to −0.17)*	<0.001	−0.07 (−0.17 to 0.03)	0.196	−0.28 (−0.38 to −0.17)*	<0.001
White (reference)	−	−	−	−	−	−
Socioeconomic position, Market Research Society's classification system						
AB	0.37 (0.33–0.40)*	<0.001	0.32 (0.28−0.36)*	<0.001	0.27 (0.23−0.31)*	<0.001
C1	0.26 (0.23–0.30)*	<0.001	0.19 (0.15−0.23)*	<0.001	0.19 (0.16−0.23)*	<0.001
C2	0.11 (0.08–0.15)*	<0.001	0.08 (0.04−0.12)*	<0.001	0.09 (0.06−0.13)*	<0.001
DE (reference)	−	−	−	−	−	−
Familiarity with mental health						
Self	0.88 (0.83–0.93)*	<0.001	0.79 (0.74−0.85)*	<0.001	0.86 (0.81−0.90)*	<0.001
Other	0.55 (0.52–0.58)*	<0.001	0.45 (0.41−0.48)*	<0.001	0.56 (0.53−0.59)*	<0.001
None (reference)	−	−	−	−	−	−
Region						
North-East	0.28 (0.21–0.35)*	<0.001	0.09 (0.02−0.17)*	0.015	0.18 (0.11−0.26)*	<0.001
North-West	0.22 (0.17–0.27)*	<0.001	0.09 (0.03−0.14)*	0.001	0.22 (0.17−0.27)*	<0.001
York and Humber	0.30 (0.24–0.35)*	<0.001	0.16 (0.10−0.22)*	<0.001	0.26 (0.20−0.32)*	<0.001
East Midlands	0.19 (0.13–0.24)*	<0.001	0.10 (0.04−0.17)*	0.002	0.20 (0.14−0.25)*	<0.001
West Midlands	0.18 (0.13–0.23)*	<0.001	0.07 (0.02−0.13)*	0.011	0.18 (0.13−0.24)*	<0.001
East	0.21 (0.16–0.26)*	<0.001	0.10 (0.04−0.15)*	0.001	0.17 (0.12−0.23)*	<0.001
South-East	0.16 (0.12–0.21)*	<0.001	0.16 (0.00−0.11)*	0.033	0.12 (0.07−0.17)*	<0.001
South-West	0.27 (0.22–0.32)*	<0.001	0.12 (0.06−0.18)*	<0.001	0.16 (0.11−0.22)*	<0.001
London (reference)	−	−	−	−	−	−

CAMI, Community Attitudes toward the Mentally Ill scale; MAKS, Mental Health Knowledge Schedule; RIBS, Reported and Intended Behaviour Scale.

* Significant at *P* < 0.05 level.

We also examined changes in CAMI ‘Prejudice and Exclusion’ and ‘Tolerance and Support for Community Care’ subscale scores (see Supplementary Table 1). Scores over time are presented in Supplementary Fig. 3. Scores on the ‘Prejudice and Exclusion’ subscale showed continued increases to 2021, but in 2023, scores returned to 2019 levels, showing only a 0.1% improvement since that time (*P* = 0.949). Since 2019, scores on the ‘Tolerance and Support for Community Care’ subscale decreased by 7.1% (*P* < 0.001).

### Stigma-related knowledge

Changes in MAKS scores (marginal estimates of the standardised scores by year) are presented in Fig. 2. Between 2009 and 2019, there were improvements in MAKS scores (see Table 2). Since 2019, MAKS scores have decreased by 7.8% (*P* < 0.001) and results from 2023 no longer differ from 2009 scores (*P* = 0.153). Interactions emerged between year and age (*P* = 0.012), class (*P* = 0.021) and region (*P* = 0.010) (see Supplementary Figs 4–6). Specifically, since 2019, MAKS scores have declined in people aged under 25 years and aged 45–64 years; in people from the AB and C2 class groups; and in the North-East, North-West, South-East and South-West of England.

### Desire for social distance

Figure 2 illustrates the change over time in total RIBS Intended Behaviour subscale scores (marginal estimates of the standardised scores). Significant improvements in scores were seen between 2009 and 2019 (Table 2). Since 2019, scores have decreased by 10.2% (*P* < 0.001) and results from 2023 no longer differ from 2009 scores (*P* = 0.294). Interactions emerged between year and socioeconomic position (*P* = 0.017) and government region (*P* < 0.001) (see Supplementary Figs 7 and 8). Declines in scores were seen in the AB and C2 socioeconomic group and in South-East England since 2019.

### Comparison with BSAS 2007 and 2015 data

**Fig. 3:**
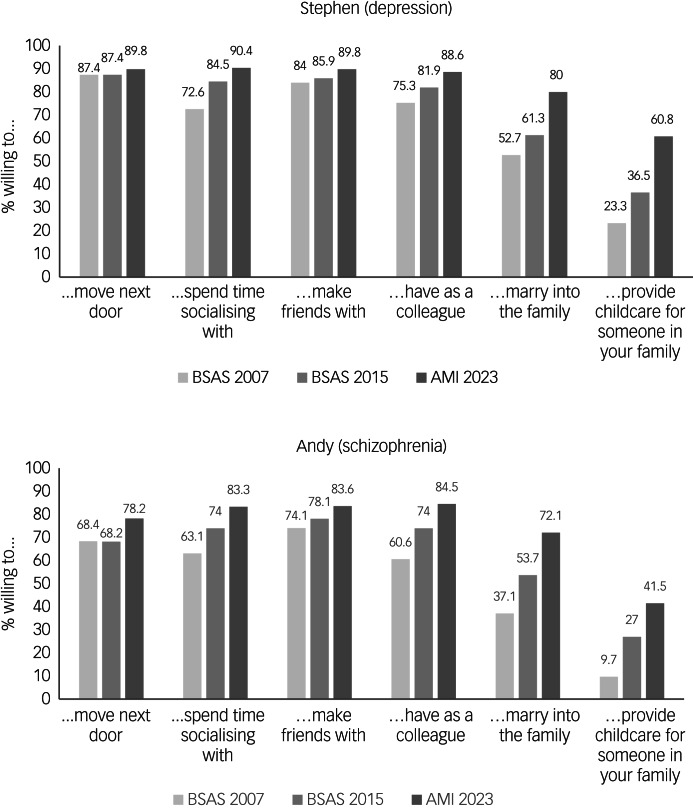
Responses to depression and schizophrenia vignettes in 2007 (BSAS), 2015 (BSAS) and 2023 (AMI). AMI, Attitudes to Mental Illness survey; BSAS, British Social Attitudes Survey.

Sample characteristics for BSAS respondents in England are provided in Supplementary Table 2 (2007 *N* = 610, 2015 *N* = 1865). Responses to each vignette item for depression and anxiety are presented in Fig. 3. Gradual increases in willingness to interact with both Stephen (depression) and Andy (schizophrenia) can be seen across all domains. Adjusted logistic regressions (Supplementary Table 3) revealed that these increases were significant for all scenarios relating to schizophrenia, and most of the scenarios relating to depression, with the exception of ‘move next door to Stephen’, which did not change significantly. In both cases, the most pronounced increases were seen in willingness to ‘have Andy/Stephen provide childcare for someone in your family’ (depression 2023: odds ratio 5.39, 95% CI 3.74–7.7; schizophrenia 2023: odds ratio 6.42, 95% CI 4.04–10.20).

In terms of workplace discrimination, proportions of participant responses from the BSAS in 2015 and the AMI in 2023 are presented in Supplementary Table 4. Adjusted logistic regressions (Supplementary Table 5) revealed that, compared with people in 2015, people in 2023 were more likely to agree that people with depression and schizophrenia are just as likely to be promoted. This change was most pronounced for schizophrenia (odds ratio 2.47, 95% CI 1.97–3.11). Moreover, people in 2023 were more likely to agree that having depression or schizophrenia should not make a difference to likelihood of promotion.

### Exploration of desire for social distance

**Fig. 4:**
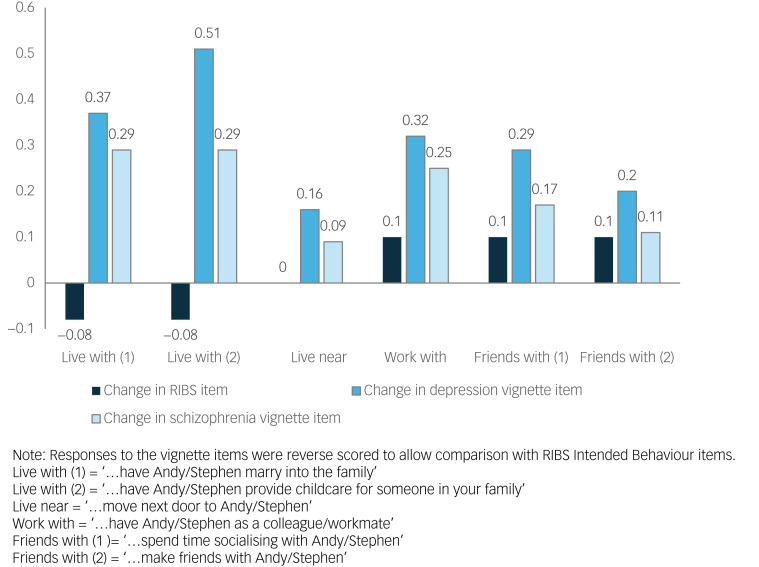
Change in RIBS Intended Behaviour subscale and vignette scores between 2015 and 2023. RIBS, Reported and Intended Behaviour Scale.

To explore why we observed both decreases in RIBS Intended Behaviour subscale scores and greater willingness to have contact based on the vignettes representing individuals with symptoms of depression and schizophrenia, we graphically compared items from the RIBS Intended Behaviour subscale and the vignettes that tapped into similar aspects of life. As both the RIBS Intended Behaviour subscale and the 2015 and 2023 vignettes were responded to on a five-point Likert scale, we could calculate change scores by subtracting mean responses to each RIBS Intended Behaviour subscale and vignette item in 2015 from responses in 2023 (the 2007 BSAS vignettes could not be included as the four-item response scale precluded the calculation of change scores). These change scores are presented in Fig. 4. Results indicate that RIBS Intended Behaviour subscale items concerning living with or near people with mental illness decreased or remained the same over this period, and items relating to working and being friends with people with mental illness increased marginally. Change scores for the vignettes suggest that there have been considerable increases in willingness to live, work and be friends with people with mental illness, particularly in relation to depression.

## Discussion

Our results show a complicated set of changes over time. Consistent with the surveys undertaken during the course of the Every Mind Matters campaign from 2019 to 2022,^14^ the stigma measures using questions about mental illness or mental health problems show an increase in stigma over this period following the improvements seen between 2008 and 2019, such that although attitudes are still more positive since 2008, stigma-related knowledge and desire for social distance began to decline before the end of Time to Change and are the same as in 2009. In contrast, the questions based on vignettes of men with depression or schizophrenia showed reduced desire for social distance since 2007, which is in line with findings in Australia from 2003/2004 to 2011.^29^ However, regarding schizophrenia, this contrasts to findings from 2006 to 2018 in the USA, where desire for social distance (measured with vignettes) has changed little since 2006; indeed, they suggest an increase, consistent with an increased perception of dangerousness.^17^ Questions regarding workplace treatment showed more support for and expectation of fair workplace treatment, for someone with experience of depression and schizophrenia since 2015. Although a greater proportion of people think that medical history should make a difference in relation to promotion for someone with schizophrenia compared with depression and diabetes, views regarding each condition have converged.

Consistent with this, the measure of stigma in relation to mental illness in general that showed least deterioration is the Prejudice and Exclusion subscale of the CAMI, which also reflects awareness of rights and values of non-discrimination. In contrast, the reduction in the Tolerance and Support for Community Care subscale score may reflect reduced support for government spending on mental healthcare at a time when personal taxation as a percentage of national income is at its highest since 1948.

The decline in stigma-related knowledge since 2019 reveals more therapeutic pessimism and less confidence in being able to help those with mental illness. This may reflect the greater difficulties in access to mental healthcare during and since the COVID-19 pandemic, as well as in self-management of mental health resulting from reduced access to salutogenic processes during lockdowns and inflation.

The contrast between the negative changes in the RIBS Intended Behaviour subscale and the positive changes in response to the vignette questions is harder to explain, as the questions are similar. One interpretation is that the AMI survey sample report generally more positive attitudes up to 2019 than those covered by the BSAS, because of different sampling and/or data collection methods. However, both the BSAS 2015 and AMI survey up to 2019 were conducted face to face, a method that may result in more socially desirable responses.^30^ Hence, although the more negative attitudes found in 2021 and 2023 compared with previous AMI results could be interpreted as resulting from this change in methods, this does not explain the positive change seen between face-to-face responses to the depression and schizophrenia vignettes in 2015 and online responses to the same questions in 2023. Further, the increase in desire for social distance measures on the RIBS Intended Behaviour subscale after March 2020 during the evaluation of Every Mind Matters was observed with an online survey throughout.

We should therefore consider whether there has been a change in how people respond to vignettes about individuals, such that less desire for social distance is expressed, whereas the RIBS Intended Behaviour subscale questions about mental illness in general among groups of people elicit increasingly negative responses since 2019. The former may reflect a more lasting impact of Time to Change, which promoted supportive contact with family, friends and colleagues experiencing a mental health problem;^31^ vignettes about an individual that use their first name create a sense of familiarity, which may generate more empathy. Further, although causality was not directly addressed during the programme, the messaging during Time to Change that anyone can experience mental health problems may have affected beliefs about heritability, and hence the question about someone with mental illness marrying into the family that was linked to the vignette. The responses to the RIBS Intended Behaviour subscale may reflect a greater desire to avoid others who are unknown, which is consistent with the decline in support for community-based care. Although fear of contagion may have influenced responses in 2021, this seems less likely for 2023. However, responses to the RIBS Intended Behaviour subscale might be more affected than the vignette questions, and for longer, by personality changes among adults identified later during the pandemic. These include reductions in agreeableness and conscientiousness, although these data are from the USA.^32^ In summary, the contrasting changes in direction may reflect the fact that vignettes versus questions about contact without vignettes capture different aspects of stigma – that against known individuals versus that toward groups of unknown people.

### Strengths and limitations

We calculated sampling errors even though a quota sample was used, which violates some statistical assumptions but allowed us to calculate results as if the data were from a probability sample. Although probability sampling has been used to measure single aspects of stigma in England at one time point,^19,23^ no current epidemiological survey has allowed repeated assessment of multiple aspects of stigma. Further, the lack of data for the BSAS items in 2019 means we cannot ascertain whether these attitudes have also worsened since 2019, after improving further after 2015. However, the analysis used nationally representative datasets and the demographic associations for attitudes are consistent with those found using the Health Survey for England 2014 data.^23,33^ Although the patterns seen in the data make it plausible that changes in mental health stigma can be somewhat attributed to the Time to Change campaign, definitive causality cannot be established. Although we included a significant number of confounders in our analyses, it is possible there were unmeasured confounders that may bias our results. A change in data collection method (i.e. face to face up to 2019, followed by paper- and web-based completion in 2021 and 2023) may partially explain increases in stigma seen from 2021 resulting from reduced likelihood of socially desirable responses. However, positive change (e.g. responses to vignettes, workplace attitudes) alongside the increases in stigma indicates that the change in data collection method probably did not significantly affect results. A further limitation concerns the use of mental health vignettes, which may not fully represent real-world scenarios. However, these vignettes were based on established Diagnostic and Statistical Manual of Mental Disorders (DSM) criteria, and therefore accurately reflect the main symptoms of the mental health conditions they depict.

### Implications

Our results reinforce the multifaceted nature of mental illness-related stigma and the importance of measuring them, including in relation to common versus less common disorders, discrimination where legislation is relevant and intended interpersonal behaviour with versus without vignettes of named individuals. We predict that the aspects of stigma that worsened since 2019 will improve if and when economic conditions and access to treatment for common mental disorders improve. However, it remains to be seen whether the improvements seen over the course of Time to Change can be regained in the absence of such a programme.

## Supporting information

Ronaldson and Henderson supplementary material Ronaldson and Henderson supplementary material

## Data Availability

The data that support the findings of this study are available from the corresponding author, A.R., upon reasonable request.
