# Supplementary material for: Investigating changes in mental illness stigma and discrimination after the Time to Change programme in England
Source: BJPsych Open. 2024 Nov 6;10(6):e199. doi: 10.1192/bjo.2024.801 (PMC11698152; doi:10.1192/bjo.2024.801)
Supplement: Ronaldson and Henderson supplementary material [file S2056472424008019sup001.docx]

**Supplementary Material**

| **Table S1.** Multiple linear regression analyses of predictors of CAMI ‘Prejudice/Exclusion’ and ‘Tolerance and Support for Community Care’ subscales | | | | |
| --- | --- | --- | --- | --- |
| Predictors | CAMI: Prejudice/Exclusion | | CAMI: Tolerance and support for community care | |
|  | Standardised effect size (95% CI) | P value | Standardised effect size (95% CI) | P value |
| Year |  |  |  |  |
| 2023 | 0.29 (0.23 to 0.36) | <0.001 | 0.10 (0.02 to 0.18) | 0.013 |
| 2021 | 0.35 (0.28 to 0.41) | <0.001 | 0.21 (0.14 to 0.29) | <0.001 |
| 2019 | 0.29 (0.23 to 0.35) | <0.001 | 0.28 (0.21 to 0.35) | <0.001 |
| 2017 | 0.22 (0.16 to 0.28) | <0.001 | 0.22 (0.15 to 0.29) | <0.001 |
| 2016 | 0.19 (0.12 to 0.25) | <0.001 | 0.27 (0.19 to 0.34) | <0.001 |
| 2015 | 0.13 (0.07 to 0.19) | <0.001 | 0.23 (0.16 to 0.30) | <0.001 |
| 2014 | 0.13 (0.07 to 0.20) | <0.001 | 0.19 (0.12 to 0.26) | <0.001 |
| 2013 | 0.03 (-0.03 to 0.09) | 0.402 | 0.13 (0.06 to 0.20) | <0.001 |
| 2012 | -0.02 (-0.08 to 0.04) | 0.531 | 0.13 (0.06 to 0.20) | <0.001 |
| 2011 | -0.06 (-0.012 to 0.01) | 0.070 | 0.11 (0.04 to 0.18) | 0.003 |
| 2010 | 0.01 (-0.05 to 0.07) | 0.776 | 0.13 (0.06 to 0.20) | <0.001 |
| 2009 | -0.07 (-0.14 to -0.01) | 0.021 | 0.09 (0.02 to 0.16) | 0.011 |
| 2008 (ref) | - | - | - | - |
| Gender |  |  |  |  |
| Female | 0.17 (0.15 to 0.20) | <0.001 | 0.12 (0.10 to 0.15) | <0.001 |
| Male (ref) | - | - | - | - |
| Age |  |  |  |  |
| 16-24 | 0.20 (0.16 to 0.24) | <0.001 | -0.21 (-0.26 to -0.17) | <0.001 |
| 25-44 | 0.24 (0.21 to 0.27) | <0.001 | -0.08 (-0.11 to -0.04) | <0.001 |
| 45-64 | 0.29 (0.25 to 0.32) | <0.001 | 0.05 (0.02 to 0.09) | 0.005 |
| 65+ (ref) | - | - | - | - |
| Ethnicity |  |  |  |  |
| Asian | -0.50 (-0.55 to -0.45) | <0.001 | -0.22 (-0.27 to -0.16) | <0.001 |
| Black | -0.37 (-0.43 to -0.30) | <0.001 | -0.23 (-0.31 to -0.16) | <0.001 |
| Other | -0.28 (-0.37 to -0.19) | <0.001 | -0.19 (-0.29 to -0.09) | <0.001 |
| White (ref) | - | - | - | - |
| Socioeconomic position |  |  |  |  |
| AB | 0.41 (0.37 to 0.44) | <0.001 | 0.22 (0.18 to 0.26) | <0.001 |
| C1 | 0.29 (0.26 to 0.33) | <0.001 | 0.16 (0.12 to 0.19) | <0.001 |
| C2 | 0.10 (0.07 to 0.14) | <0.001 | 0.10 (0.06 to 0.14) | <0.001 |
| DE (ref) | - | - | - | - |
| Familiarity with mental health |  |  |  |  |
| Self | 0.86 (0.81 to 0.91) | <0.001 | 0.69 (0.64 to 0.74) | <0.001 |
| Other | 0.56 (0.53 to 0.59) | <0.001 | 0.40 (0.37 to 0.43) | <0.001 |
| None (ref) | - | - | - | - |
| Region |  |  |  |  |
| North East | 0.26 (0.19 to 0.32) | <0.001 | 0.24 (0.16 to 0.31) | <0.001 |
| North West | 0.20 (0.15 to 0.25) | <0.001 | 0.19 (0.14 to 0.25) | <0.001 |
| York and Hum | 0.26 (0.20 to 0.31) | <0.001 | 0.28 (0.22 to 0.34) | <0.001 |
| East Midlands | 0.15 (0.10 to 0.21) | <0.001 | 0.19 (0.13 to 0.25) | <0.001 |
| West Midlands | 0.19 (0.14 to 0.24) | <0.001 | 0.12 (0.07 to 0.18) | <0.001 |
| East of England | 0.19 (0.14 to 0.24) | <0.001 | 0.18 (0.12 to 0.24) | <0.001 |
| South East | 0.16 (0.12 to 0.21) | <0.001 | 0.13 (0.07 to 0.18) | <0.001 |
| South West | 0.27 (0.22 to 0.32) | <0.001 | 0.20 (0.25 to 0.26) | <0.001 |
| London (ref) | - | - | - | - |

**Figure S1.** Marginal estimates of year*age group interaction (95% CIs) for mental health related attitudes (CAMI scores)

**Figure S2.** Marginal estimates of year*region interaction (95% CIs) for mental health related attitudes (CAMI scores)

**Figure S3.** Marginal estimates of CAMI subscale scores by year (95% CIs)

**Figure S4.** Marginal estimates of year*age group interaction (95% CIs) for mental health related knowledge (MAKS scores)

**Figure S5.** Marginal estimates of year*class interaction (95% CIs) for mental health related knowledge (MAKS scores)

**Figure S6.** Marginal estimates of year*region interaction (95% CIs) for mental health related knowledge (MAKS scores)

**Figure S7.** Marginal estimates of year*socioeconomic position interaction (95% CIs) for mental health related knowledge (RIBS IB scores)

**Figure S8.** Marginal estimates of year*region interaction (95% CIs) for mental health related knowledge (RIBS IB scores)

| **Table S2.** BSAS sample characteristics for participants residing in England (2007 and 2015) | | |
| --- | --- | --- |
|  | 2007 (N=610) | 2015 (N=1865) |
|  | N(%) | N(%) |
| Age group |  |  |
|  | Missing N=0 | Missing N=5 |
| *16-24y* | 36 (5.9) | 131 (7.0) |
| *25-34y* | 108 (17.7) | 279 (15.0) |
| *35-44y* | 120 (19.7) | 334 (18.0) |
| *45-54y* | 108 (17.7) | 337 (18.1) |
| *55-64y* | 98 (16.1) | 294 (15.8) |
| *65+y* | 140 (22.9) | 485 (26.1) |
| Gender |  |  |
|  | Missing N=0 | Missing N=0 |
| *Male* | 270 (44.3) | 788 (42.2) |
| *Female* | 340 (55.7) | 1077 (57.8) |
| Ethnicity |  |  |
|  | Missing N=2 | Missing N=2 |
| *Asian* | 23 (3.8) | 111 (6.0) |
| *Black* | 16 (2.6) | 68 (3.6) |
| *Other* | 15 (2.5) | 34 (1.8) |
| *White* | 554 (91.1) | 1650 (88.6) |
| Socioeconomic position |  |  |
|  | Missing N=26 | Missing N=92 |
| *AB* | 207 (35.4) | 704 (39.7) |
| *C1* | 15 (26.5) | 369 (20.8) |
| *C2* | 129 (22.1) | 314 (17.7) |
| *DE* | 93 (15.9) | 386 (21.8) |
| Local government region |  |  |
|  | Missing N=0 | Missing N=0 |
| *North East* | 39 (6.3) | 103 (5.5) |
| *North West* | 80 (13.1) | 263 (14.1) |
| *Yorks and Humber* | 70 (11.5) | 172 (9.2) |
| *East Midlands* | 57 (9.3) | 219 (11.7) |
| *West Midlands* | 68 (11.1) | 200 (10.7) |
| *Eats of England* | 66 (10.8) | 177 (9.5) |
| *London* | 70 (11.5) | 207 (11.1) |
| *South East* | 100 (16.4) | 349 (18.7) |
| *South West* | 60 (9.8) | 175 (9.4) |

| **Table S3.** Logistic regressions assessing changes in willingness to interact with people with schizophrenia or depression between 2007 (BSAS) and 2015 (BSAS) and 2023 (AMI). Note ‘neither willing nor unwilling’ responses were removed from 2015 and 2023 to facilitate comparison with 2007 data. This has led to reduced sample sizes. | | | | | |
| --- | --- | --- | --- | --- | --- |
|  | 2007 | 2015 | | 2023 | |
| Vignette: Schizophrenia (Andy) | N | N | OR (95% CI) | N | OR (95% CI) |
| \| And now we would like you to think about how willing, or unwilling, you would be to ... \| \| --- \| |  |  |  |  |  |
| *…move next door to Andy?* | 288 | 437 | 0.92 (0.65 to 1.29) | 403 | 1.64 (1.13 to 2.37)* |
| *…spend time socializing with Andy?* | 287 | 438 | 1.56 (1.11 to 2.20)* | 413 | 2.85 (1.96 to 4.15)** |
| *…make friends with Andy?* | 290 | 438 | 1.24 (0.85 to 1.80) | 421 | 1.81 (1.21 to 2.69)* |
| *…have Andy as a colleague/workmate?* | 287 | 435 | 1.84 (1.30 to 2.60)* | 414 | 3.67 (2.50 to 5.38)** |
| *…have Andy marry into the family?* | 280 | 436 | 2.15 (1.54 to 3.00)** | 373 | 4.95 (3.44 to 7.13)** |
| *…have Andy provide childcare for someone in your family?* | 288 | 434 | 3.47 (2.18 to 5.52)** | 359 | 6.42 (4.04 to 10.20)** |
|  | 2007 | 2015 | | 2023 | |
| Vignette: Depression (Stephen) | N | N | OR (95% CI) | N | OR (95% CI) |
| \| And now we would like you to think about how willing, or unwilling, you would be to ... \| \| --- \| |  |  |  |  |  |
| *…move next door to Stephen?* | 309 | 438 | 1.07 (066 to 1.74) | 423 | 1.20 (0.72 to 2.00) |
| *…spend time socializing with Stephen?* | 307 | 440 | 2.13 (1.43 to 3.16)** | 428 | 3.71 (2.36 to 5.83)** |
| *…make friends with Stephen?* | 307 | 440 | 1.16 (0.75 to 1.81) | 433 | 1.71 (1.06 to 2.79)* |
| *…have Stephen as a colleague/workmate?* | 308 | 437 | 1.59 (1.07 to 2.35)* | 421 | 2.53 (1.64 to 3.91)** |
| *…have Stephen marry into the family?* | 300 | 437 | 1.63 (1.17 to 2.26)* | 389 | 4.07 (2.79 to 5.93)** |
| *…have Stephen provide childcare for someone in your family?* | 305 | 435 | 1.94 (1.37 to 2.75)** | 375 | 5.39 (3.74 to 7.76)** |
| Note: BSAS 2007 acts as reference category  Logistic regression adjusted for age, sex, ethnicity, socioeconomic position, and government region  AMI=Attitudes to Mental Illness; BSAS: British Social Attitudes Survey; CI=confidence interval; OR=odds ratio  *p<0.05, **p<0.001 | | | | | |

| **Table S4.** Changes in attitudes to mental health in the workplace measured in the British Social Attitudes Survey (BSAS 2015) and ‘Time to Change’ (2023) | | |
| --- | --- | --- |
|  | *BSAS 2015*  *N=2140* | *Time to Change 2023*  *N=1638* |
| *Views on promotion prospects* | 2015 | 2023 |
| **Depression** |  |  |
| % just as likely as anyone else to be promoted | 16.5 | 25.5 |
| % slightly less likely | 47.5 | 44.6 |
| % much less likely | 36.0 | 29.9 |
| % medical history definitely/probably should make a difference | 37.5 | 15.4 |
| **Schizophrenia** |  |  |
| % just as likely as anyone else to be promoted | 8.3 | 17.7 |
| % slightly less likely | 32.1 | 36.4 |
| % much less likely | 59.6 | 45.8 |
| % medical history definitely/probably should make a difference | 50.0 | 20.4 |
| **Diabetes** |  |  |
| % just as likely as anyone else to be promoted | 56.9 | 63.8 |
| % slightly less likely | 35.4 | 31.0 |
| % much less likely | 7.6 | 5.2 |
| % medical history definitely/probably should make a difference | 25.2 | 13.5 |

| **Table S5.** Logistic regressions assessing change in attitudes towards promotions in the workplace for people with depression, schizophrenia, or diabetes between 2015 (BSAS) and 2023 (AMI) | | | | | |
| --- | --- | --- | --- | --- | --- |
|  |  | Just as likely to be promoted | | Condition definitely/probably should not make a difference | |
|  |  | *OR (95% CI)* | *P value* | *OR (95% CI)* | *P value* |
| Depression | BSAS 2015 | Ref |  | Ref |  |
|  | AMI 2023 | 1.76 (1.49 to 2.11) | <0.001 | 1.48 (1.23 to 1.81) | <0.001 |
| Schizophrenia | BSAS 2015 | Ref |  | Ref |  |
|  | AMI 2023 | 2.47 (1.97 to 3.11) | <0.001 | 1.50 (1.25 to 1.81) | <0.001 |
| Diabetes | BSAS 2015 | Ref |  | Ref |  |
|  | AMI 2023 | 1.31 (1.13 to 1.52) | <0.001 | 1.38 (1.13 to 1.68) | 0.001 |
| AMI=Attitudes to Mental Illness; BSAS: British Social Attitudes Survey; CI=confidence interval; OR=odds ratio | | | | | |
